# Macrophage-derived exosome promotes regulatory T cell differentiation in malignant pleural effusion

**DOI:** 10.3389/fimmu.2023.1161375

**Published:** 2023-04-18

**Authors:** Ming-Ming Shao, Xue-Bin Pei, Qing-Yu Chen, Feng Wang, Zhen Wang, Kan Zhai

**Affiliations:** Department of Respiratory and Critical Care Medicine, Beijing Institute of Respiratory Medicine and Beijing Chao-Yang Hospital, Capital Medical University, Beijing, China

**Keywords:** malignant pleural effusion, macrophages, exosomes, miR-4443, Treg

## Abstract

**Introduction:**

Tumor-associated macrophages are one of the key components of the tumor microenvironment. The immunomodulatory activity and function of macrophages in malignant pleural effusion (MPE), a special tumor metastasis microenvironment, have not been clearly defined.

**Methods:**

MPE-based single-cell RNA sequencing data was used to characterize macrophages. Subsequently, the regulatory effect of macrophages and their secreted exosomes on T cells was verified by experiments. Next, miRNA microarray was used to analyze differentially expressed miRNAs in MPE and benign pleural effusion, and data from The Cancer Genome Atlas (TCGA) was used to evaluate the correlation between miRNAs and patient survival.

**Results:**

Single-cell RNA sequencing data showed macrophages were mainly M2 polarized in MPE and had higher exosome secretion function compared with those in blood. We found that exosomes released from macrophages could promote the differentiation of naïve T cells into Treg cells in MPE. We detected differential expression miRNAs in macrophage-derived exosomes between MPE and benign pleural effusion by miRNA microarray and found that miR-4443 was significantly overexpressed in MPE exosomes. Gene functional enrichment analysis showed that the target genes of miR-4443 were involved in the regulation of protein kinase B signaling and lipid biosynthetic process.

**Conclusions:**

Taken together, these results reveal that exosomes mediate the intercellular communication between macrophages and T cells, yielding an immunosuppressive environment for MPE. miR-4443 expressed by macrophages, but not total miR-4443, might serve as a prognostic marker in patients with metastatic lung cancer.

## Introduction

Malignant pleural effusion (MPE) is commonly encountered among cancer patients and a common cause of mortality worldwide. The appearance of MPE implies systemic spread of disease and reduction of life expectancy and quality (1–3). Given that both tumor cells and host immune cells, including lymphocytes and myeloid cells, are enriched in MPE are enriched in MPE and that the collaboration is responsible for the formation and development of MPE (4–9). Macrophage and CD4^+^ T cells were the dominant cell types. Previous studies demonstrated that multiple subtypes of helper T (Th) cells, including regulatory T (Treg), Th1, Th17, Th9, Th22 cells and activated naive B cells also played important roles in the pathogenesis of MPE (5, 10–12). MPE therefore is a good disease model for exploring immune environment of advanced cancer.

As a pivotal component of host immune cells, macrophages play a vital role in immune response. Macrophages existing in tumor tissues or populating in the tumor microenvironment are termed as tumor-associated macrophages that display pro-tumor functions by enhancing the malignancy of cancer cells, including promotion of proliferation and angiogenesis, regulation of metabolism and suppression of adaptive immunity, and therefore correlate with poor prognosis in human cancers (13, 14). Giannou et al. reported that removal of macrophages results in reduced volume of MPE, indicating that macrophages are required for MPE formation (15). Macrophages are able to interact with T lymphocytes to mediate immune suppression. Li et al. demonstrated that macrophages could impair T cell function in MPE by releasing TGF-β (16). Other studies have reported that macrophages can release exosomes, which are single-membrane vesicles with a diameter of 30-200 nm (17). Exosomes are enriched in various bioactive components, including proteins, lipids, DNAs, mRNAs and microRNAs (miRNAs). Once released, exosomes can deliver their cargo into recipient cells to regulate cell function (18, 19). However, the function of exosomes in MPE remains unknown.

Regulatory T (Treg) cells, as major suppressor T cells, play essential roles in immune system. Our previous study has reported that the Treg cells were increased in MPE and inhibited the proliferation of responding T cells (10). It was further demonstrated that Treg cells were recruited by the chemokine CCL22 (20). However, the upstream mechanisms of Treg cells differentiation have not been clarified yet.

In this study, we investigated the communication between macrophages and T cells mediated by exosomes in MPE. We found that exosomes from macrophages could promote the differentiation of Treg cells *in vitro*. Microarray data showed that several miRNAs were dysregulated in exosomes from MPE as compared with those from benign pleural effusion (BPE). Gene function enrichment analysis suggested that miR-4443, which was upregulation in MPE, might promote Treg cell differentiation by targeting genes related with lipid biosynthetic process and protein kinase B signaling. Moreover, we analyzed the effect of miR-4443 expression on patient survival in the cancer genome atlas (TCGA) lung adenocarcinoma data and found that among metastatic patients, patients with high macrophage miR-4443 expression had worse survival. Our results suggest a previously unrecognized immunoregulation activity and the underlying mechanism for macrophages to favor the differentiation of Treg cells.

## Materials and methods

### Patients and specimens

The study protocol was approved by the Institutional Review Boards of Beijing Chao-Yang Hospital, Capital Medical University (2018-ke-327), Nanning Fourth People’s Hospital (201928), Tongji Medical College, Huazhong University of Science and Technology (2019-IEC-S809) and Wuhan Pulmonary Hospital (2019–1). From June 2018 to December 2019, a total of 78 patients with definite diagnosis of pleural effusions were enrolled in our study. All participants signed written informed consent. MPE was obtained from 59 patients, including 45 subjects were adenocarcinoma, 10 were squamous cell carcinoma, 2 were small cell lung cancer, and 2 were mesothelioma. BPE was collected form 19 patients, with 12 subjects were tuberculosis, 5 were pneumonia, and 2 were heart failure. **Supplementary Table 1** presents the clinical characteristics of these patients. A diagnosis of MPE was established by the appearance of malignant cells in pleural effusion and/or on closed pleural biopsy samples. Tuberculous effusion was diagnosed if *Mycobacterium tuberculosis* were positive in biopsy specimen, or if the granulomas were found in pleural tissue. Patients enrolled in this research were all HIV-negative and had no history of corticosteroids, antineoplastic therapy, or any other drugs known to affect immune status.

### Differential expressed genes and gene set variation analysis

The “FindAllMarkers” and “FindMarkers” function in R package was used to identify the differential expression genes. The parameter indicators for differential expression genes were: detected in at least 30% of the cells in the target cluster, Wilcoxon test *P* value less than 0.05 and log fold change of expression above 0.25. Heatmap of gene expression was drawn by DoHeatmap. Signaling pathway enrichment analysis of differential expression genes was performed with GSEA function in clusterProfiler package (3.16.1). C5 gene ontology (biological process) signatures gene sets were downloaded from MSigDB (http://software.broadinstitute.org/gsea/msigdb/genesets.jsp). Significantly different scores across clusters were determined by ANOVA with an FDR corrected *P* value < 0.05.

### Cell sorting and coculture

Naïve CD4^+^ T cells and macrophages were separated from pleural effusion using naïve CD4^+^ T cell isolation kit II (Miltenyi Biotec, Bergisch Gladbach, Germany) and CD14 microbeads (Miltenyi Biotec), respectively. The purity of isolated cells was > 90%, and verified by flow cytometry. Naïve CD4^+^ T cells (2 × 10^6^/mL) were suspended in complete RPMI 1640 medium with 10% fetal bovine serum (FBS) and cultured on 96-well plates coated with anti-CD3 (5 μg/mL) and anti-CD28 (2 μg/mL). Naïve CD4^+^ T cells and macrophages were cocultured for 5 days at a 1:1 ratio. For the inhibition of exosome releasing, GW4869 (Sigma-Aldrich, St Louis, MO) dissolved in dimethyl sulfoxide (Sigma-Aldrich) was added into the cell suspension at a concentration of 10 μM.

For the coculture of naïve CD4^+^ T cells and exosomes, naïve CD4^+^ T cells were prestimulated with anti-CD3 (5 μg/mL) and anti-CD28 (2 μg/mL) mAbs for 3 days. After coculture with exosomes (50 μg/ml) derived from macrophages for 3 days, naïve CD4^+^ T were stimulated with Phorbol-12-Myristate-13-Acetate (50 ng/mL, Sigma-Aldrich), ionomycin (1 μg/mL, Sigma-Aldrich), and Brefeldin A (10 μg/mL, Enzo Life Sciences, Farmingdale, NY) for 5 hours and harvested for further analysis.

### Isolation and characterization of exosomes

To obtain macrophage-derived exosomes, macrophages were cultured in complete RPMI 1640 containing 10% exosome-free FBS. FBS was previously centrifugated at 120,000 g for 18 hours at 4 °C to remove bovine exosome (21). After 72 hours, the media was collected and exosomes were isolated using Ribo™ Exosome Isolation Reagent (for cell culture media, Ribobio, Guangzhou, China) following the manufacturer’s protocol. In brief, the conditioned media of macrophages was centrifuged at 2,000 g for 30 minutes to remove cells and debris. Supernatants were added to exosome isolation reagent and incubated overnight, followed by centrifugation at 1,500 g for 30 minutes at 4°C. The pellets resuspended in PBS were prepared for follow-up experiments.

For visualization of exosomes, the purified pellets diluted in PBS were deposited on formvar carbon-coated nickel grids, stained with 2% uranyl acetate, and observed under a JEM-1200 transmission electron microscope. The particle size and concentration were determined using a ZetaView instrument (Particle Metrix, Meerbusch, Germany).

### Transfer of PKH26-labelled exosomes

The purified exosomes were stained with a PKH26 red fluorescent cell linker kit (Sigma-Aldrich). After incubation with PKH26, exosomes were washed using 100 KDa filters (Amicon Ultra-4 mL, Millipore, Darmstadt, Germany) to eradicate redundant lipid dye. For the cellular uptake of exosome analysis, naïve CD4^+^ T cells were preactivated under stimulation with anti-CD3 (5 μg/mL) and anti-CD28 (2 μg/mL) mAbs for 24 hours and cocultured with labelled exosomes for another 24 hours. The results were detected under a confocal microscope.

### Immunofluorescence

Isolated mononuclear cells from MPE were cultured on slides in complete RPMI 1640 medium containing 10% FBS overnight. The slides were washed and fixed in 4% paraformaldehyde at room temperature for 15 minutes. After permeabilizing for 15 minutes in 0.1% Triton X-100, slides were washed followed by blocking in 10% donkey serum for 30 minutes, and then incubated overnight with primary Abs (CD14, 1:200; CD206, 1:250; CD86, 1:250; all from Abcam) at 4°C. Then, the slides were washed again and incubated for 1 hour with secondary Abs conjugated to Alexa Fluor 488 (1:100, AntGene Biotech, Wuhan, China) or 594 (1:100, AntGene) at room temperature in the dark. For nuclei staining, slides were washed and incubated with DAPI (Sigma-Aldrich) for 10 minutes. Antifade solution was added, and slides were dried and observed under an imaging fluorescence microscope (Olympus, Tokyo, Japan).

### microarray analysis of miRNA

miRNA expression profile of macrophage-derived exosomes from MPE and BPE was conducted with an Agilent Human miRNA microarray (8*60K, Design ID: 070156), which was performed by OE Biotech (Shanghai, China) Limited Company. miRNeasy Serum/Plasma Kit (Qiagen, Germany) was used to extract exosomal RNA, and 100 ng RNA from each specimen was used for microarray analysis. The raw data have been deposited in the OMIX, China National Center for Bioinformation/Beijing Institute of Genomics, Chinese Academy of Sciences (https://ngdc.cncb.ac.cn/omix, accession no.OMIX726) (22).

### RNA extraction, reverse transcription and qRT-PCR

TRIzol Reagent (Thermo, Waltham, MA, USA) was used to extract total RNA from exosomes and cells following the manufacturer’s protocol. cDNA was synthesized using a PrimeScript™RT reagent Kit with gDNA Eraser (Takara, Dalian, China) for mRNA analysis and using miRNA first strand cDNA synthesis (Sangon Biotech, Shanghai, China) for miRNA analysis, respectively. qRT-PCR was conducted with SYBR Green I master (Roche, Basel, Switzerland) and analyzed in a LightCycler 480 (Roche). miRNA expression levels were normalized to small nuclear RNA U6. Each sample was performed in triplicate and the fold change was analyzed by the 2^-ΔΔCT^ method. Information about the primers is provided in **Supplementary Table 2**.

### Transfection with miRNA mimic

Naïve CD4^+^ T cells were isolated and cultured (2 × 10^6^/mL) on anti-CD3 (5 μg/mL) and anti-CD28 (2 μg/mL)-coated 96-well plates in complete RPMI 1640 medium with 10% FBS under the Treg condition (IL-2 [5 ng/mL] and TGF-β [10 ng/mL]; both from Peprotech, Rocky Hill, NJ). Cells were transfected with miR-4443 mimic or negative control (100 nM; Ribobio) by using Lipofectamine RNAiMAX Reagent (Life Technologies, Carlsbad, CA) according to the manufacturer’s recommendations. Cells were then cultured for 72 hours before further analysis.

### Flow cytometry

Cell suspension from pleural effusion and blood samples were treated with Lymphocyte Separation Medium (MP Biomedicals), and mononuclear cells were separated by density gradient centrifugations within 1 hour. Then cells were collected and stained for the following flow cytometric analysis.

These Abs for flow cytometry, including anti-CD45, -CD45RA, -CD14, -CD206, -CD86, -CD3, -CD4, -CD8, -CD25, -IFN-γ, -IL-17, -IL-22, -IL-27, -IL-4, -IL-9, and -FOXP3 mAbs, were purchased from Thermo or BD Biosciences (San Diego, CA). Briefly, for the cell surface receptors including CD45, CD45RA, CD14, CD206, CD86, CD3, CD4, CD8, CD25 cells were incubated with the corresponding Abs for 15 min in dark. For the intracellular cytokines and transcription factors, such as IFN-γ, IL-17, IL-22, IL-27, IL-4, IL-9, and FOXP3, cells were conducted with fixation/permeabilization solution (BD Biosciences) for 30 min, washed by permeabilization buffer (BD Biosciences) and the stained with Abs for 20 min in dark. Then cells were washed and fixed in 4% paraformaldehyde. Gating of cells was as follows: macrophages: FSC/SSC/CD45^+^/CD14^+^; Th1 cells: FSC/SSC/CD3^+^/CD8^–^/IFN-γ^+^; Treg cells: FSC/SSC/CD3^+^/CD8^–^/CD25^+^/FOXP3^+^; Th17 cells: FSC/SSC/CD3^+^/CD8^–^/IL-17^+^; Th2 cells: FSC/SSC/CD3^+^/CD8^–^/IL-4^+^; Th9 cells: FSC/SSC/CD3^+^/CD8^–^/IL-9^+^; Th22 cells: FSC/SSC/CD3^+^/CD8^–^/IL-22^+^; Th27 cells: FSC/SSC/CD3^+^/CD8^–^/IL-27^+^. Results were performed on a FACS Canto II (BD Biosciences, CA, USA), and analyzed using FCS Express 5 software (*De Novo* Software, Los Angeles, CA).

### TCGA data sources and processing

mRNA sequencing data, tumor node metastasis classification and survival information of LUAD patients were obtained from the UCSC Xena (https://xena.ucsc.edu/). Gene expression data was converted into Transcripts Per Kilobase Million (TPM), and log2 (TPM + 0.01) was applied for subsequent analysis. The impact of miR-4443 expression on the overall survival of LUAD patients was evaluated by Kaplan-Meier analysis. CIBERSORT algorithm was used to calculate the proportion of immune cell types according to the description of Newman et al. (23). To eliminate the influence of different immune cell proportions, we divided the expression of miR-4443 in the TCGA data by the cell fraction estimated by CIBERSORT for normalization.

### Statistical analysis

Data are shown as mean ± SEM. Independent experiments were carried out at least in triplicate. We performed Student’s *t* test, Fisher’s exact test, Mann-Whitney test or one-way ANOVA followed by Bonferroni’s test for two or multiple groups, as appropriate. Results were analyzed using GraphPad Prism 7 (San Diego, CA, USA) Software. Statistical differences were assumed if the two-tailed value of *P* < 0.05.

## Results

### Macrophages were characterized by M2 phenotype in MPE

**Figure 1:**
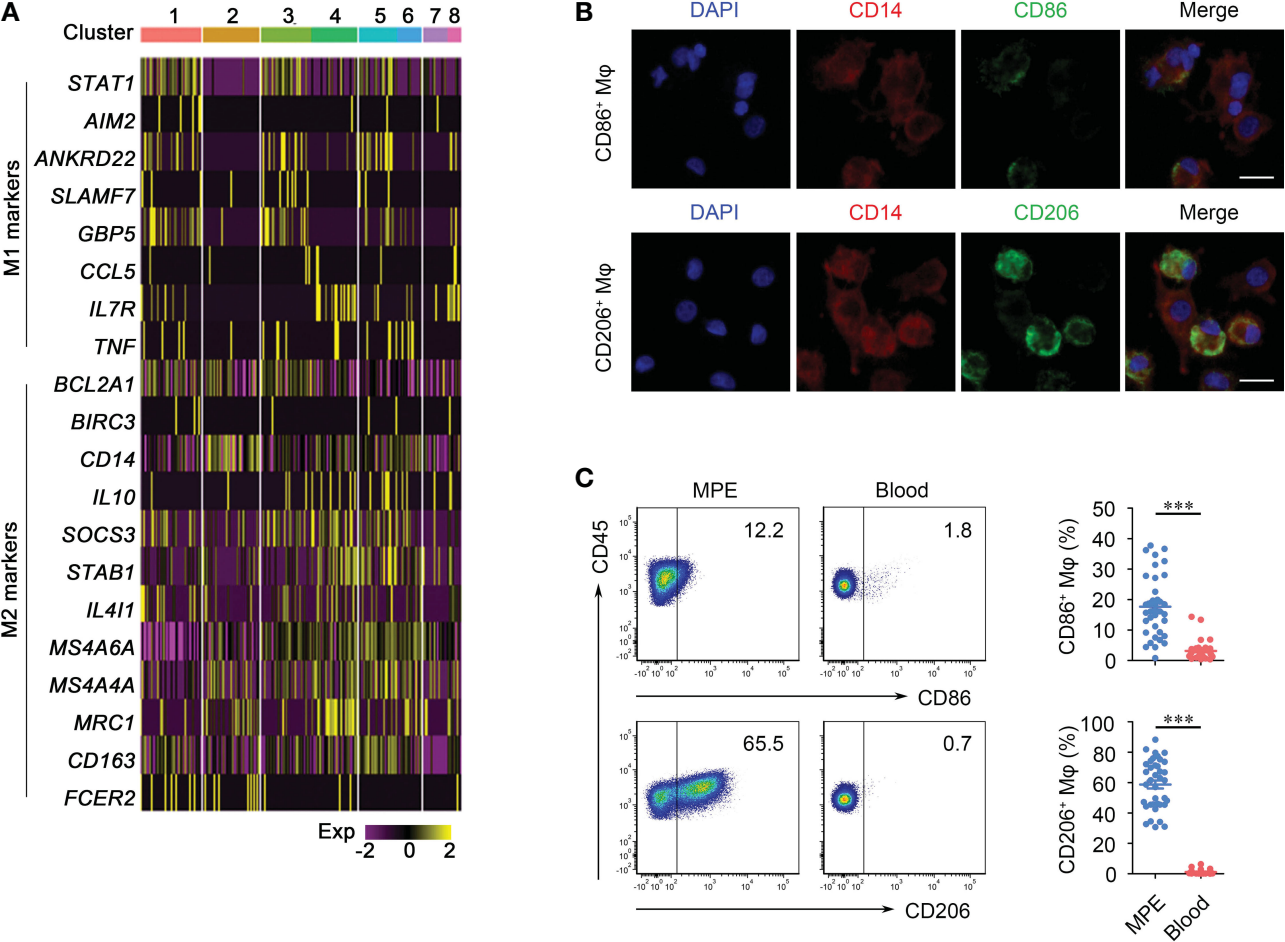
Phenotypes of macrophages (Mφs) in malignant pleural effusion (MPE). **(A)** Heatmap shows the marker genes of M1 and M2 pattern of Mφs in sub-clusters. **(B)** Immunofluorescence images of CD86^+^Mφ (upper panel) and CD206^+^Mφ (bottom panel). Representative images are shown; 200 × magnification; scale bar, 20 μm. Red, CD14; green, CD86/CD206; blue, DAPI. **(C)** Representative flow cytometry plots of CD86^+^Mφ and CD206^+^Mφ (gated on CD45^+^CD14^+^ Mφ) in MPE and the corresponding blood (left panel). Comparisons of percentages of CD86^+^Mφ and CD206^+^Mφ are shown in MPE and blood (right panel) (n = 38). Comparisons were made using Student’s *t* test; ***P < 0.001.

Macrophages was a major population in MPE, and we identified 7211 macrophages from our single cell sequencing data of five MPE samples. In order to reveal the internal structure and potential functional subtypes of these macrophage populations, we performed unsupervised clustering and classified them into eight stable clusters (**Supplementary Figure 1A**). Tumor-associated macrophages are often thought to polarize towards an inflammatory M1 or pro-tumor M2 macrophages, depending on their environmental stimuli (24, 25). Although these eight cell sub-clusters had their own expression characteristics, such as M-1 cluster highly expressed SPP1 and CXCL8 genes and M-5 cluster highly expressed C1QA and C1QB genes (**Supplementary Figure 1B**), The expression of M2 polarization marker genes was generally higher than that of M1 in all these eight sub-clusters (**Figure 1A**). Consistent with the foregoing results, double immunofluorescence staining demonstrated that macrophages in MPE mainly expressed M2-related markers (CD206) and rarely expressed M1-related markers (CD86) (**Figure 1B**). Flow cytometry analysis further confirmed that macrophages were predominantly of a M2 phenotype. Although the proportions of M1 macrophages (CD86^+^) and M2 (CD206^+^) macrophages in MPE were both significantly higher than those in peripheral blood, the fold increase of M2 macrophages was much higher than that of M1 macrophages (**Figure 1C**).

### Macrophages regulated Th1 cells and Treg cells differentiation in MPE

**Figure 2:**
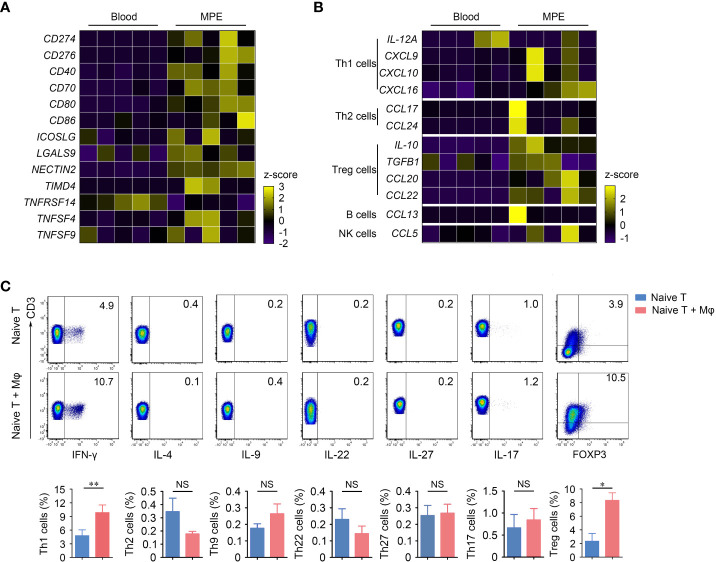
Mφs in MPE interact with T cells and promote the differentiation of Th1 cells and Treg cells. **(A)** Heatmap shows the lymphocyte costimulatory molecule profile of Mφs in MPE and blood. **(B)** Heatmap shows the cytokine and chemokine profile of Mφs in MPE and blood. **(C)** Representative flow cytometry plots of Th1 cells, Th2 cells, Th9 cells, Th22 cells, Th27 cells, Th17 cells and Treg cells are shown from one of three independent experiments (upper panel). Comparisons of percentages of these cells are shown in the bottom panel. Data are presented as mean ± SEM. Comparisons were made using Student’s *t* test. *P < 0.05, **P < 0.01. NS, not significant.

In the tumor microenvironment, macrophages are central regulators of T cell activation and are involved in each step. During lymphocyte activation, costimulatory signals are critical for the generation of the effective immune response, and macrophages can secrete costimulatory signals and cytokines required for lymphocyte activation. So we first detect lymphocyte costimulatory molecular profile of macrophages in MPE and blood single cell RNA sequencing data (26). Most of the lymphocyte costimulatory molecules, such as CD274, CD276, CD40, CD70, CD80, CD86, ICOSLG, LGALS9, NECTIN2, TIMD4, TNFRSF14, TNFSF4 and TNFSF9, had higher expression levels in MPE than in blood (**Figure 2A**), indicating that macrophages in MPE have a stronger function of activating lymphocytes. To identify the subtype of lymphocytes that macrophages are most likely to correlate with in MPE, we further investigated the lymphocyte-related cytokines and chemokine profiles of macrophages in MPE and blood. The cytokines and chemokines acting on Th1 cells (IL-12A, CXCL9, CXCL10 and CXCL16), Th2 cells (CCL17 and CCL24) and Treg cells (IL-10, TGFB1, CCL20 and CCL22) were more clearly enriched in macrophages compared with B cells or NK cells in MPE (**Figure 2B**) (25, 27), indicating that macrophages in MPE might interact with CD4^+^ T cells. To verify this result, we cocultured macrophages and naïve CD4^+^ T cells isolated from MPE under the medium condition at a ratio of 1:1 for 5 days and found that macrophages had the capability to promote naïve CD4^+^ T cells into Th1 cells and Treg cells. Besides, macrophages had no effects on the differentiation of Th2, Th9, Th22, Th27, or Th17 cells (**Figure 2C**). Overall, the above results indicate that macrophages might promote the differentiation of Th1 cells and Treg cells in MPE.

### The ability of macrophages to secrete exosomes was enhanced in MPE

**Figure 3:**
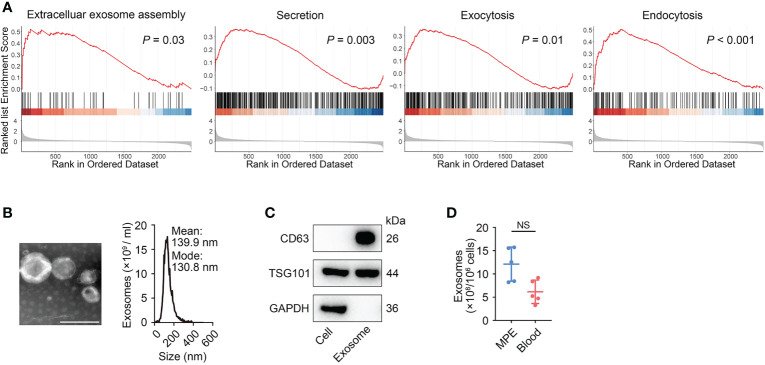
Mφs could secrete exosomes. **(A)** GSEA analyses revealed that Mφs in MPE exhibited higher pathway activity for “extracellular exosomes assembly”, “secretion”, “exocytosis”, and “endocytosis” as compared to blood. All *P*-values were calculated by permutation test according to the standard GSEA procedure. **(B)** A representative transmission electron microscopy image of purified exosomes (left panel) and the characterization of purified exosomes by using nanoparticle tracking (right panel). Scale bar, 100 nm. **(C)** Western blot analysis for CD63, TSG101, and GAPDH in the whole cell lysate and purified exosomes. **(D)** Total exosomes in Mφs purified from MPE (n = 5) and blood (n = 5). Data are presented as mean ± SEM. Mann-Whitney test U; NS, not significant.

In addition to traditionally providing costimulatory signals for T cell activation, we also propose to investigate whether there are other pathways by which macrophages regulate lymphocytes. We performed GSVA analysis on differential expression genes of macrophages between MPE with peripheral blood and identified that in addition to the immune response related functions such as response to interferon gamma, cytokine-mediated signaling pathway and inflammatory responses, macrophages in MPE also displayed strong signals of extracellular exosomes assembly, secretion, exocytosis and endocytosis (**Figure 3A** and **Supplementary Figure 2**) (19, 28). The enhancement of these signaling pathways suggested that macrophages might secrete more exosomes than those in blood. To verify the ability of macrophages to secrete exosomes, we cultured macrophages sorted from MPE in exosome-free medium for 72 hours and collected supernatant to isolate exosomes using Ribo™ Exosome Isolation Reagent. We observed the morphology and structure of isolated vesicles using electron microscopy and determined vesicle size and concentration using ZetaView particle tracker. **Figure 3B** showed that vesicles purified from cultured macrophage supernatants presented a goblet-shaped membrane structure with a diameter of about 130 nm, conforming to the morphology and size of typical exosomes. Western blot showed that the conventional exosome biomarkers CD63 and TSG101 could be detected on the surface of the isolated vesicles (**Figure 3C**) (29). These results indicated that macrophages extracted from MPE indeed had the ability to secrete exosomes. We next isolated equal numbers of macrophages from MPE and blood samples and extracted exosomes to compare the difference in exosome content between the two groups. The results showed that the number of exosomes from MPE samples was higher than that from blood samples (*P* = 0.056), but the P value did not reach statistical significance due to the small sample size (n = 5) (**Figure 3D**). Taken together, these data suggested that macrophages in MPE were able to secrete more exosomes.

### Exosomes derived from macrophages induce the differentiation of Treg cells

**Figure 4:**
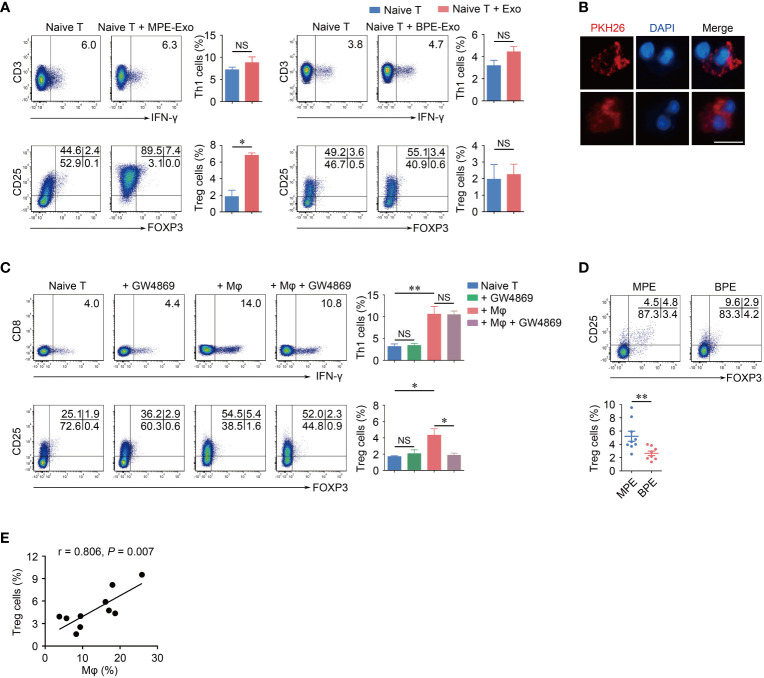
Exosomes from Mφs in MPE promote Treg cells differentiation *in vitro*. **(A)** Naïve CD4^+^ T cells were cocultured in the presence of control or purified exosomes from MPE Mφs (MPE-Exo) or BPE Mφs (BPE-Exo) under stimulation with anti-CD3 and anti-CD28 mAbs for 3 days. The representative flow cytometry plots of Th1 cells and Treg cells are shown from three independent experiments. Comparisons of percentages of Th1 cells and Treg cells are shown. **(B)** Immunofluorescence images of exosomes (red) taken up by CD4^+^ T cells after treatment with PKH26-labeled exosomes. 400 × magnification; Scale bar, 10 μm. **(C)** Naïve CD4^+^ T cells were cocultured with Mφs treated with GW4869 (10 μM) or control in the presence of anti-CD3 and anti-CD28 mAbs for 5 days. The representative flow cytometry plots of Th1 cells and Treg cells are shown from one of three independent experiments. Comparisons of percentages of Th1 cells and Tregs are shown. **(D)** Representative flow cytometry plots of Treg cells in MPE and BPE. Comparisons of percentages of Treg cells are shown in MPE and BPE. **(E)** The percentages of Treg cells were correlated with Mφs in MPE. Correlations were analyzed by Spearman’s rank correlation coefficients. Data are presented as mean ± SEM. Comparisons were made using Student’s *t* test. *P < 0.05, **P < 0.01, NS, not significant.

Exosomes are an important medium for intercellular communication, so we investigate whether the T cell differentiation-promoting effect of macrophages in MPE is mediated by exosomes. We cultured naïve CD4^+^ T cells under stimulation with anti-CD3 and anti-CD28 mAbs in the presence of exosomes derived from macrophages in MPE (MPE-Exo). Notably, after coculture with MPE-Exo for 72 hours, the differentiation of Treg cells was significantly increased (*P* < 0.05); whereas the differentiation of Th1 cells was not affected (*P* = 0.153) (**Figure 4A**). Clinically, in addition to MPE, benign pleural effusion (BPE) due to inflammation or malnutrition is another common type of pleural effusion in which the macrophages are predominantly M1 polarized. We cocultured naïve CD4^+^ T cells with exosomes from the same amounts of macrophages in BPE (BPE-Exo), and found that BPE-Exo did not affect the differentiation of either Treg cells or Th1 cells (**Figure 4A**). To confirm that T cells are able to take up exosomes, we cultured pre-stimulated naïve CD4^+^ T cells in the presence of PKH26-labeled exosomes for 24 hours, and then detected them by using confocal microscopy. It was noted that exosomes isolated from macrophages could be taken up by naïve CD4^+^ T cells, supporting the interaction between exosomes and naïve CD4^+^ T cells (**Figure 4B**). We blocked exosome releasing by treating macrophages with GW4869, which was reported to be a specific N-SMase inhibitor and could suppress exosome formation and release from cells (30). After GW4869 treatment, macrophages failed to promote the differentiation of Treg cells, but not Th1 cells (**Figure 4C**). In addition, we detected the proportions of Treg cells in both MPE and BPE and found that the proportion of Treg cells in MPE was significantly higher than that in BPE (*P* < 0.01) (**Figure 4D**). Consistently, we also noticed that Treg cells showed a positive correlation with macrophages in MPE (*r* = 0.806, *P* = 0.007) (**Figure 4E**). In conclusion, these findings indicated that macrophages could induce Treg cells differentiation by transmitting exosomes in MPE.

### miR-4443 was increased in macrophage-derived exosomes

**Figure 5:**
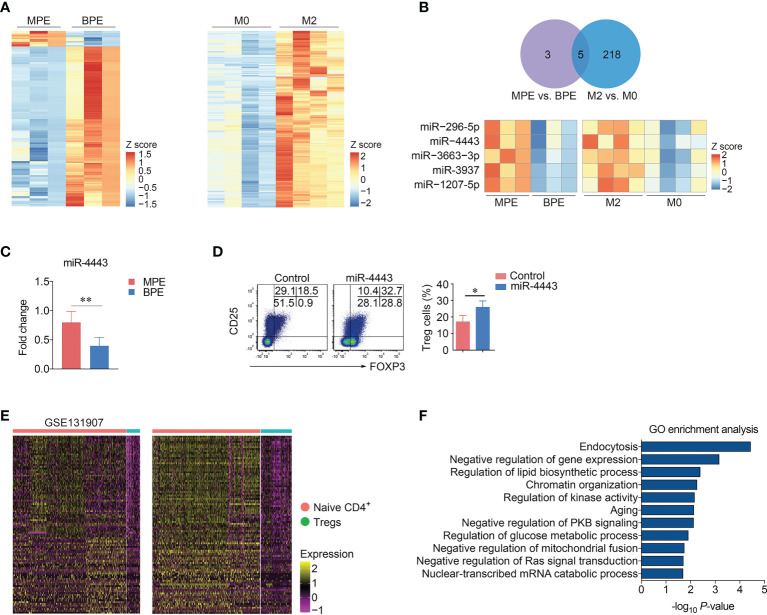
miR-4443 induced the differentiation of Treg cells. **(A)** Heatmaps showed the miRNA expression profile of the exosomes from Mφs in MPE and benign pleural effusion (BPE) (each n = 3) (left panel) or in M0 and M2 Mφs downloaded from GSE97467. **(B)** miR-296-5p, miR-4443, miR-3663-3p, miR-3937 and miR-1207-5p were up-regulation in both two data sets. **(C)** Expression of miR-4443 in the exosomes from MPE and BPE detected by RT-qPCR (each n = 8). Data are presented as mean ± SEM. Comparisons were made using Student’s *t* test. **P < 0.01, NS, not significant. **(D)** Naïve CD4^+^ T cells were transfected with miR-4443 mimic and negative control under Treg condition (IL-2 and TGF-β) for 3 days. Representative flow cytometry plots of Treg cells (left panel) are displayed from four independent experiments. Comparison of percentages of Treg cells (right panel) is shown. Data are presented as mean ± SEM. Comparisons were made using Student’s *t* test. *P < 0.05. **(E)** The expression of target genes of miR-4443 in Naive CD4^+^ T cells and Treg cells. **(F)** GO enrichment analysis of the target genes of miR-4443.

Exosomes transfer specific mRNA and/or miRNA to targeted cells, thus altering their gene expression and function (17). To understand how exosomal miRNAs affected the differentiation of Treg cells, we applied an Agilent miRNA microarray to profile miRNAs in MPE-Exo and BPE-Exo. A total of 824 miRNAs were detected in macrophage-derived exosomes, of which 44 miRNAs were differential expression in MPE-Exo compared with those in BPE-Exo. As macrophages in MPE are dominated by M2 polarization, we downloaded miRNA microarray data of M2 and M0 macrophage-derived exosomes from the GEO database for further validation (GSE97467) (**Figure 5A**). The expression of miR-296-5p, miR-1207-5p, miR-3663-3p, miR-3937 and miR-4443 was significantly up-regulation in both data sets (**Figure 5B**). We further validated the expression of these five differential miRNAs in an additional 8 pairs of samples using qRT-PCR. However, only miR-4443 was significantly increased in MPE-Exo compared with BPE-Exo (*P* < 0.05) (**Figure 5C** and **Supplementary Figure 3**). To evaluate the functionality of miRNAs in exosomes, naïve CD4^+^ T cells were transfected with miR-4443 mimic or negative control and cultured *in vitro* under Treg cells condition for 72 hours. We found that after transfecting with miR-4443 mimic, the differentiation of Treg cells was significantly increased (*P* < 0.05, **Figure 5D**).

miRNAs often negatively regulate gene expression at the post-transcriptional level through complementary binding to mRNA. To evaluate the functions of miRNAs in exosomes, we selected mRNAs predicted in five online miRNA target prediction databases (miRDB, http://mirdb.org/; miRWalk, http://mirwalk.umm.uni-heidelberg.de/; RNA22, https://cm.jefferson.edu/rna22/; TargetScan, https://www.targetscan.org/vert_80/; RNAInter, http://www.rnainter.org/) as potential miRNA-acting genes. A total of 443 genes were simultaneously predicted as target genes of miR-4443 in these five databases. We next investigated the expression of these 443 genes in Naive CD4^+^ T cells and Treg cells in MPE single-cell RNA sequencing data and screened 107 genes whose expression in Treg cells was lower than that in Naive CD4^+^ T cells in both our and GSE97467 data (**Figure 5E**). GSVA analysis showed that these low-expressed genes were mainly involved in negative regulation of gene expression, regulation of lipid biosynthetic process and negative regulation of protein kinase B signaling, etc (**Figure 5F**).

**Figure 6:**
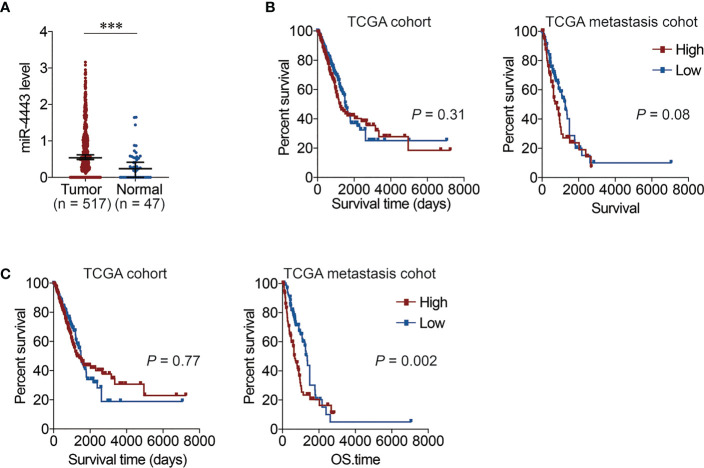
The relationship between the expression of miR-4443 with lung cancer progression in TCGA data. **(A)**. The expression of miR-4443 in TCGA lung adenocarcinoma (LUAD) data. Comparisons were made using Student’s *t* test. ***P < 0.001. **(B)**. The Kaplan-Meier overall survival curves of TCGA LUAD patients grouped by miR-4443 in all patients (left panel) or metastasis patients (right panel). The high and low group are divided by the median value of miR-4443. **(C)**. The Kaplan-Meier overall survival curves of TCGA LUAD patients grouped by miR-4443 in all patients (left panel) or metastasis patients (right panel). The high and low group are divided by the median value of miR-4443 with normalization by the proportion of macrophages calculated using CIBERSORT (lower panel). The significant was calculated using the two-sided log-rank test.

Finally, we explored the potential relationship between the expression levels of miR-4443 with lung cancer progression in TCGA data. The expression of miR-4443 was significantly up-regulated in tumor tissues compared with normal tissues (**Figure 6A**). It was noted that there was no association with the expression of miR-4443 and the survival of either all LUAD patients or metastatic patient (**Figure 6B**). However, after normalization for the macrophage fractions, patients with high expression of miR-4443 showed a shorter overall survival in metastatic patients (**Figure 6C**). The above results suggested that for patients with metastatic lung cancer, miR-4443 in macrophages could be used as a prognostic marker.

## Discussion

Immune cells enriched in MPE, such as helper T cells, B cells and macrophages, have been intensively discussed. However, the pathogenesis and local immune response of MPE remains uncertain. In the present study, our data revealed a new mechanism by which macrophages interact with CD4^+^ T cells. Compared with BPE, macrophages in MPE promote Treg cells differentiation by transmitting lower exosomal miR-4443.

Macrophages are one type of main immune cells infiltrating into MPE and present a wide range of M1/M2 spectrum (31). Consistent with previous studies (32–34), our present study confirmed that macrophages in MPE were characterized by M2-polarized phenotype. Although the immunoregulatory function of macrophages have been illustrated in most tumor models, the underlying role of macrophages in MPE remains to be clarified.

Within tumor microenvironment, the crosstalk between macrophages and T cells was commonly reported. Macrophages usually exhibit pro-tumor activity, with promotion of Th2 response, induction of Treg cells and recruitment of Treg cells into tumor sites (25, 35, 36). Besides, macrophages can also drive T cells towards Th1 response by secreting pro-inflammatory cytokines in human colorectal cancer (37). Since the functions of macrophages on regulating T cells were varied, we intended to investigate the relationship between macrophages and CD4^+^ T cells in MPE. Our results showed that macrophages in MPE interacted with CD4^+^ T cells and promoted the differentiation of Th1 cells and Treg cells.

Exosomes carry numerous components and can deliver them into targeted cells to regulate cellular function (18). By means of exosomes, CD14^+^ macrophages promoted CD4^+^ T-cell activation-induced cell death resistance in Crohn’s disease (38). Furthermore, macrophages could transfer miR-29a-3p and miR-21-5p into CD4^+^ T cells, thus suppressing STAT3 expression and inducing a Treg/Th17 imbalance in epithelial ovarian cancer (39). All these data suggest that macrophages may regulate T cells functions by transmitting exosomes, thus participating in physiological and pathological process. Similarity, our data also revealed that exosomes were abundant in macrophages and could be taken up by CD4^+^ T cells, supporting the interaction between CD4^+^ T cells and macrophage-derived exosomes. We further demonstrated that macrophage-derived exosomes promoted the differentiation of Treg cells. However, the differentiation of Th1 cells was not induced by exosomes, which will be further analyzed. These results indicated that exosomes isolated from macrophages play an important role in promoting Treg cells differentiation.

Treg cells are indispensable in various diseases such as cancer, inflammation and autoimmunity, for their critical role in the maintenance of immune homeostasis and tolerance (40). Tregs were a critical subset of T cells that played an important role in peripheral tolerance, autoimmunity, and tumor immunity by exercising inhibitory functions in T-cell-mediated immune responses (41). Tregs not only inhibited the antitumor immunity mediated by effector T cells, but also released IL-17 and transferred into T follicular regulatory cells to disrupt Tfh response, thus promoting the occurrence and development of tumors (42). The enrichment of Tregs was also reported in MPE, which was consistent with our findings, suggesting that tumor microenvironment could induce more Tregs to accumulate in the pleural cavity (43). In addition, a higher proportion of Tregs was also associated with poorer overall survival in MPE patients (44). These phenotypic characteristic of Tregs in the tumor environment might provide a basis for them to be potential targets for enhancing anti-tumor immune response. Certain miRNAs such as miR-17-92 cluster, miR-17 and miR-146a participate in Treg cells development and immune function (45–47). However, there are few reports on the relationship between exosomal miRNAs and Treg cells. To further explain the underlying mechanism involved in exosome-mediated Treg cells differentiation, we explored miRNA expression profiles in exosomes from MPE and BPE. By analyzing these differentially expressed miRNAs, miR-4443 emerged as a candidate of interest. Unlike effector T cells, Treg cells select fatty acid oxidation as the primary metabolic type (48). During fatty acid synthesis, its metabolic intermediates inhibit the occurrence of fatty acid oxidation, ensuring that fatty acid synthesis and fatty acid oxidation do not occur simultaneously (49). We speculate that after Naive T cells in MPE ingested macrophage-derived exosomes containing higher levels of miR-4443, the expression of fatty acid synthesis-related genes decreased, which could lead to enhanced fatty acid oxidation and promote Treg cell differentiation. Previous studies have also found that miR-4443 can exist in exosomes and exert its biological effect. In breast tumor, cancer cells delivered exosomal miR-4443 to stromal cells of the primary tumor and impaired TIMP2 to promote liver metastasis (50). Exosomal miR-4443 promotes cisplatin resistance in non-small cell lung carcinoma by regulating FSP1 m6A modification-mediated ferroptosis (51). Drusco et al. found that miR-4443 was most significantly elevated in serum exosomes from glioma patients compared with healthy controls, but its expression was not associated with patients survival (52). Since the lack of serum miRNA expression results, we used TCGA data to compare the expression of miR-4443 in tumor tissues and adjacent normal tissues of LUAD patients and found that miR-4443 was significantly overexpression in tumor tissues. Although survival data from TCGA patients also showed that miR-4443 expression was not associated with prognosis in LUAD patients, when miR-4443 expression values were normalized by the macrophage fractions, patients with high miR-4443 expression in metastatic LUAD patients showed a shorter survival time.

Previous studies have reported multiple functions of miR-4443 in different disease microenvironments. In patients with Graves disease, increased expression of miR-4443 induced CD4^+^ T cell dysfunction by targeting TRAF4 (53). In papillary thyroid carcinoma, head and neck squamous cell carcinoma, and non-small cell lung cancer, miR-4443 was involved in the regulation of tumor cell proliferation, migration, or resistance to chemotherapeutics (54–56). Our study found that in MPE, a special microenvironment of lung cancer metastasis, macrophages with M2-polarized phenotype secreted miR-4443-rich exosomes, which could be taken up by T cells and promoted Treg cell differentiation. We synthesized the results of multiple miRNA prediction and validation databases to initially identify a series of potential target genes of miR-4443, which were involved in signaling pathways such as positive regulation of lipid biosynthetic process, negative regulation of gene expression and negative regulation of protein kinase B signaling, which all have been reported to be associated with Treg cell differentiation (57, 58). The molecular mechanism of miR-4443 regulating Tregs and the detailed immunophenotypic changes still needed to be discussed in our subsequent studies. In addition to miRNA, there were a large number of other non-coding RNAs and proteins in macrophage exosomes, and the role of these components in the intercellular communication between macrophages and Tregs remained to be explored.

In conclusion, we demonstrate that in MPE, macrophages delivered exosomes with enrichment of miR-4443 to naive CD4^+^ T cells, thereby promoting Treg cell differentiation. Our study revealed a new insight into macrophage-mediated regulation of T cells and provides a new approach for targeted therapy of MPE.

## Data availability statement

The datasets presented in this study can be found in online repositories. The names of the repository/repositories and accession number(s) can be found below: https://ngdc.cncb.ac.cn/omix, OMIX726.

## Ethics statement

The study protocol was approved by the Institutional Review Boards of Beijing Chao-Yang Hospital, Capital Medical University (2018-ke-327), Nanning Fourth People’s Hospital (201928), Tongji Medical College, Huazhong University of Science and Technology (2019-IEC-S809) and Wuhan Pulmonary Hospital (2019–1). Research in this study has been performed in accordance with the Declaration of Helsinki and has been approved by an appropriate ethics committee. We confirm that all methods were performed in accordance with the relevant guidelines and regulations. Informed consent was obtained from all study participants.

## Author contributions

M-MS and KZ designed and analyzed the data, and drafted the manuscript. X-BP, M-MS and Q-YC performed cellular experiments. ZW and FW recruited the subjects. KZ conceived the idea, supervised the study, designed experiments, analyzed data, critically revised the paper and guarantee the study’s integrity. All authors read and approved the final manuscript.
