# Supplementary material for: Macrophage-derived exosome promotes regulatory T cell differentiation in malignant pleural effusion
Source: Front Immunol. 2023 Apr 18;14:1161375. doi: 10.3389/fimmu.2023.1161375 (PMC10151820; doi:10.3389/fimmu.2023.1161375)
Supplement: Supplementary file 1 [file DataSheet_1.docx]

**Supplementary Table 1. Demographics of patients with pleural effusion include in single-cell RNA sequencing, microarray, quantitative real-time PCR and flow cytometry experiments.**

| Variable | Single-cell RNA sequencing | Microarray experiment | | Validation cohort (miRNA) | | Flow cytometry experiment | |
| --- | --- | --- | --- | --- | --- | --- | --- |
|  | MPE | MPE | BPE | MPE | BPE | MPE | BPE |
| Number | 5 | 3 | 3 | 5 | 5 | 46 | 11 |
| Age, yr | 65.0 ± 2.6 | 77.3 ± 1.2 | 49.7 ± 12.5 | 65.6 ± 4.0 | 43.8 ± 7.7 | 66.7 ± 1.9 | 53.8 ± 7.9 |
| Sex, Male/Female | 4/1 | 3/0 | 3/0 | 2/3 | 4/1 | 31/15 | 10/1 |
| Nucleated cell counts, ⅹ 10^9^/L | 2.9 ± 0.6 | 1.0 ± 0.5 | 3.1 ± 1.1 | 2.5 ± 1.3 | 2.7 ± 0.7 | 1.4 ± 0.6 | 2.0 ± 0.5 |
| Protein, g/L | 49.9 ± 3.2 | 42.4 ± 4.1 | 41.5 ± 8.4 | 46.5 ± 2.7 | 48.9 ± 1.3 | 39.7 ± 2.1 | 42.3 ± 3.9 |
| Glucose, mmol/L | 4.4 ± 3.5 | 9.0 ± 0.6 | 8.4 ± 2.6 | 5.2 ± 1.7 | 6.1 ± 1.2 | 6.0 ± 0.5 | 5.8 ± 0.4 |
| Lactate dehydrogenase, U/L | 924.3 ± 421.8 | 459.0 ± 132.0 | 510.0 ± 176.7 | 345.0 ± 107.6 | 297.5 ± 37.1 | 369.2 ± 61.0 | 396.3 ± 120.4 |

MPE, malignant pleural effusion; BPE, benign pleural effusion.

**Supplementary Table 2. Primer sequences used in this study.**

| Small nuclear RNA U6 | CTCGCTTCGGCAGCACA |
| --- | --- |
| Hsa-miR-296-5p | CCCCCCCTCAATCCT |
| Hsa-miR-4443 | TTGGAGGCGTGGGTTT |
| Hsa-miR-3663-3p | GCACCACACAGGCCGG |
| Hsa-miR-3937 | ACAGGCGGCTGTAGCAAT |
| Hsa-miR-1207-5p | TGGCAGGGAGGCTGGGA |
| Universal reverse | GCTGTCAACGATAGCTACCT |

**
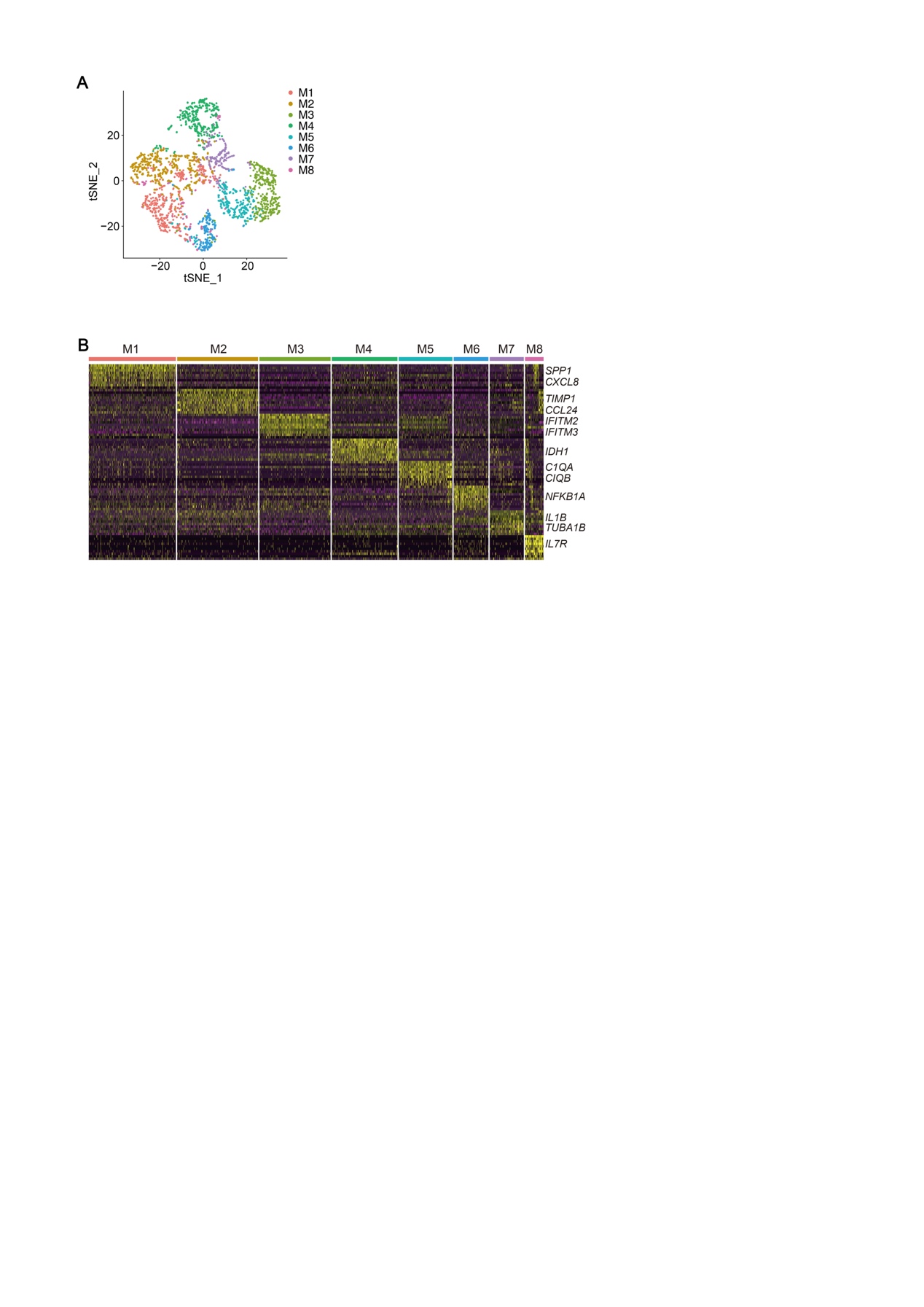
**

**Supplementary Figure 1. Dissection of macrophages of single cell RNA sequencing data from MPE.**
(**A**). Mφs in MPE could be divided into eight sub-clusters. (**B**). Heatmap shows the marker genes in eight Mφ sub-clusters.


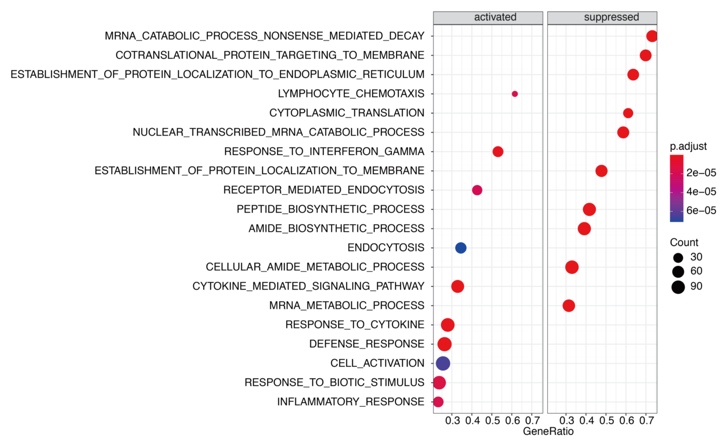


**Supplementary Figure 2. GSVA analysis on differential expression genes of macrophages between MPE with peripheral blood.**


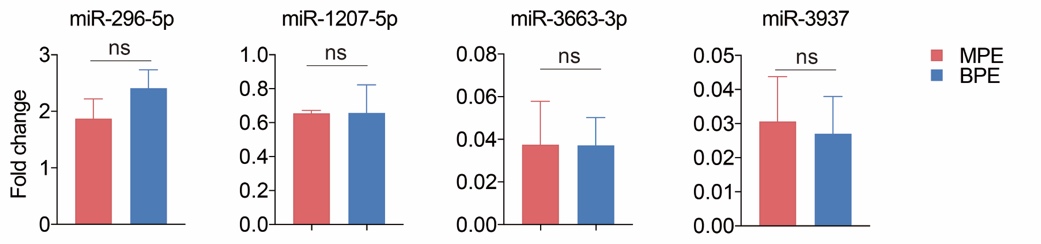


**Supplementary Figure 3.** Expression of miR-296-5p, miR-3663-3p, miR-3937 and miR-1207-5p in the exosomes from MPE and BPE detected by RT-qPCR (each n = 8). Data are presented as mean ± SEM. Comparisons were made using Student’s *t* test. NS, not significant
